# Crisis affected people with intellectual disabilities: experiences of conflict, natural hazard related disaster, and public health emergencies

**DOI:** 10.3389/fpsyt.2026.1844950

**Published:** 2026-07-06

**Authors:** Brigitte Rohwerder

**Affiliations:** Institute of Development Studies, University of Sussex, Brighton, United Kingdom

**Keywords:** conflict, crises, intellectual disabilities, natural hazard related disaster, public health emergencies

## Abstract

Across the world conflicts, natural hazard related disasters, and public health emergencies, have a disproportionate impact on the lives of people with disabilities. Such crises exacerbate their pre-existing inequalities, create new barriers, and disrupt their support structures. Their right to protection is recognised in Article 11 of the UN Convention on the Rights of Persons with Disabilities. One of the factors that changes the impact of different crises on people with disabilities is impairment type, yet there has been little specific focus on crisis affected people with intellectual disabilities. This review synthesises the available evidence about crisis affected people with intellectual disabilities’ experiences of conflicts, natural hazard related disasters, and public health emergencies. It highlights the disproportionate impact of crises that children and adults with intellectual disabilities experience, the factors which contribute to this disproportionate impact on them, and their experiences of preparedness, response, and post-crises.

## 1 Introduction

Globally, people are increasingly exposed to crises such as conflict, natural-hazard related disasters, and public health emergencies such as the Covid-19 pandemic (1). According to the Armed Conflict Location and Event Data (ACLED), over 240,000 people were killed by conflict in the past 12 months, and in 2024 almost 17,000 people were killed by natural hazard related disasters, while 14.9 million excess deaths were associated with the COVID-19 pandemic in 2020 and 2021 (2–4). UNHCR, the UN Refugee Agency, notes that in 2025 there were over 117 million people displaced by conflict and over the past decade around 250 million people displaced by natural hazarded related disasters (5). As a result, the impact of crises on people with disabilities was a focus area of the 2025 Global Disability Inclusion Report, and an accompanying background paper which reviewed available evidence about the experiences of people with disabilities in crises (6, 7).

People with disabilities were found to be disproportionally impacted by crises, which exacerbate their pre-existing inequalities and social exclusion, disrupt their support systems, and create new barriers to services and participation in society (6, 7). They face heightened risk of death, violence, abuse, as well as negative effects on their health, livelihoods, education, and participation (6–11).

Despite Article 11 of the UN Convention on the Rights of Persons with Disabilities making disability inclusion in crises a legal requirement and slow progress towards greater disability inclusion being made in relation to it, people with disabilities have often been excluded from crises preparedness and emergency or humanitarian response (7, 9, 12–14). They experience a variety of barriers including attitudinal, communication, environmental, and institutional barriers to preparedness and response, making it more challenging for them to evacuate when needed and have their basic needs met during and after crises (7, 9, 12–14).

People with disabilities are not homogenous and their experiences of poverty or institutionalisation, their age, gender, race, displacement status, geographic isolation, as well as their impairment type can affect the impact of a crisis on them (7). However, there has been little specific focus on crisis affected people with intellectual disabilities. Given the focus of this special issue on the intersectional perspectives of people with intellectual disabilities and due to the author’s interest in conflict affected people with intellectual disabilities (15), as well as the number of people with intellectual disabilities who are part of the world’s population increasingly affected by a variety of crises, this paper seeks to redress this lack of focus by synthesising the available evidence and providing an overview about what is known about the experiences of crisis-affected people with intellectual disabilities in conflict, disasters, and public health emergencies.

## 2 Methodology

**Figure 1:**
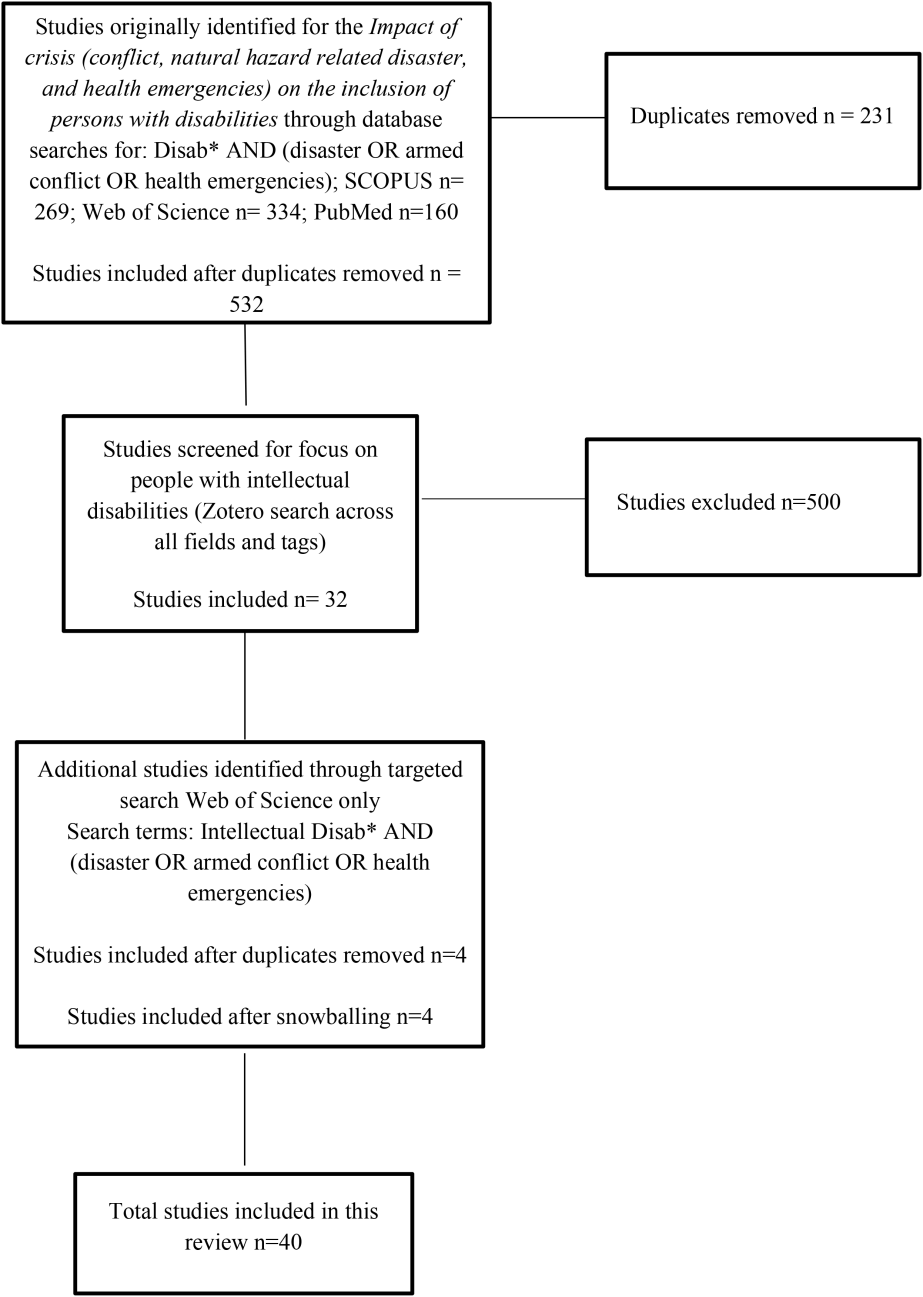
Identification and selection of studies.

The aim of this paper was to expand on a previous literature review carried out by the author for background paper for the 2025 Global Disability Inclusion Report looking at the available evidence on the *Impact of crisis (conflict, natural hazard related disaster, and health emergencies) on the inclusion of persons with disabilities* (7), and explore the impact that crises have on people with intellectual disabilities specifically. The background paper searched SCOPUS, Web of Science, and PubMed for disability in general, using the search terms *‘*Disab*AND (disaster OR “armed conflict” OR “health emergencies”)’, for publications between 2011-2024. This returned 532 academic articles, of which a search in Zotero where they were saved (for All fields and tags) found only 32 articles which were available to the author that focused on people with intellectual disabilities. As the initial search was generically for ‘disab*’, a further search of Web of Science, which had returned the most relevant results for the original search^1^, was conducted focusing specifically on ‘“Intellectual Disab*”AND (disaster OR “armed conflict” OR “health emergencies”)’ in line with the original search terms. This was to discover if any relevant articles had been missed by the generic search and did not have a date limit. As a result, a further 4 articles were included (excluding duplicates). And additional 4 articles were added through snowballing. Articles were included if they explored the experiences of people with intellectual disabilities in crises directly, or via their caregivers. The total number of papers included for this review was 40 (see **Figure 1**). Each article was thematically analysed, and the main themes relating to the experiences of crises affected people with intellectual disabilities emerging from these articles were then synthesised and the finding are outlined below.

The review may have missed relevant papers as due to time limitations, additional searches for other terms for people with intellectual disabilities, such as ‘learning disabilities’, ‘developmental delay’, etc., and for specific crisis types, such as Covid-19, as well as additional databases, were not able to be carried out.

## 3 Findings

### 3.1 Crises affected people with intellectual disabilities – themes:

#### 3.1.1 Disproportionate impact

In armed conflict, people with intellectual disabilities face increased risk of death and injury, as direct targets or indirectly (15, 16). In Nazi Germany, people with intellectual disabilities were amongst the estimated 90,000–270,000 people with disabilities killed, labelled “useless eaters” and whose lives were not deemed of value and to be worth living, while there were reports of people with Down’s Syndrome used as suicide bombers in Iraq by militants who had ‘befriended’ them and used them, disregarding their right to life (15, 16). In other cases, they have been killed because soldiers or other actors have misunderstood their behaviour as threatening or non-compliant, despite being told that they had intellectual disabilities (15, 16). During flight from conflict they may be left behind, find it harder to flee, and they face great difficulties in internally displaced persons or refugee camps (15, 17).

People with intellectual disabilities experience heightened risks and barriers during and after natural hazard related disasters and are more likely to experience poor outcomes due to communication barriers and disrupted support systems (18, 19). People with intellectual disabilities have been found to have higher mortality and injury rates in earthquakes compared to the general population, as they may struggle to understand approaching dangers, find safe areas, and protect themselves (20). Earthquakes, wildfires, and other disasters and their aftermaths lead to serious health, social, psychological and educational difficulties for children and adults with intellectual disabilities, and they struggle to cope (20–22).

People with intellectual disabilities suffered disproportionately in health outcomes and general well-being during public health emergencies, like the COVID-19 pandemic (23). A systematic review of Covid-19 related mortality found that people with intellectual disabilities were at the highest relative risk of COVID-19 mortality compared to people without disabilities^2^, due to factors such as barriers to seeking health care and information, including negative attitudes, lack of accessible information, higher risk of infection, and for some people with intellectual disabilities there may also be biological vulnerabilities (24). Other studies found a higher prevalence of comorbidities (endocrine, respiratory, and pulmonary) associated with poorer COVID-19 outcomes amongst people with intellectual disabilities (10, 25).

#### 3.1.2 Factors contributing to disproportionate impact of crises on people with intellectual disabilities

People with intellectual disabilities experience disproportionate risk in crises situations (20, 26, 27). As they experience challenges with executive function (such as planning, initiating and carrying out goal directed behaviour), memory, retaining information, adaptive behaviour, problem solving and decision-making, children and adults with intellectual disabilities are less aware of social and practical risks, which places them at additional risk in crises (10, 15, 19, 25, 26, 28, 29). Their social isolation, reduced education levels, reduced employment rates, higher poverty rates, and reduced access to health care as a result of the different barriers they face, increase their susceptibility to risk as they enter into crises from a position of less resources and weaker support structures (15, 17, 27). They are often very dependent on their families or caregivers in emergencies^3^ (17, 19). Any prior history of intra- and extra-familial abuse and neglect (because of their dependence on others, social isolation, and family stress) also affects children and adults with intellectual disabilities ability to cope with traumatic events during crises (10, 25).

Their impairment is often less visible to those responding to crises, which means they may not receive the support required to cope with crises contexts until those needs become critical and thus apparent to others, who may not understand how to meet those needs (15, 18, 26, 29–31). Other people may not understand their different communication needs, behaviour, and need for more time (19, 29–31). Those responding to crises often lack the training to work effectively with people with intellectual disabilities and to understand their needs and capacities (21, 26, 29, 30).

The stigma and discrimination people with intellectual disabilities experience in everyday life increases their risks during crises, while resource-scarcity in crises tends to exacerbate discrimination and exclusion (15, 16, 18, 25, 32). People with intellectual disabilities lives are at risk because they are deemed to have very little value (15, 16, 30). During the Covid-19 pandemic people with intellectual disabilities experienced discrimination in healthcare, and complaints were made in relation to triage protocols and Do Not Resuscitate orders which prioritized abled lives over a life with an intellectual disability, and other biases against people with intellectual disabilities in health care systems (33).

#### 3.1.3 Inclusion (or lack of) in preparedness

While there is increasing recognition of the importance of disability inclusion in crises preparedness and response (34, 35), the diversity of disability is not always recognized, which means the specific needs and different experience of people with intellectual disabilities are overlooked (15, 27, 30, 36).

Despite the importance of the involvement of both children and adults with intellectual disabilities in preparedness activities, such activities are often not inclusive of them, especially if there are stigmatizing beliefs such as being disabled means they won’t be able to take part (18, 28, 32, 36, 37). A study of 275 people with intellectual disabilities aged 10 years or older in Korea found that their level of coping skills (concepts of prevention, preparedness, response, and recovery) and knowledge in the face of emergencies or disasters was low (38). Their low levels of coping skills was attributed to their education levels and lack of inclusion in comprehensive disaster coping training (38). Only 13.5% of people with intellectual disabilities in the study had experience of comprehensive disaster response education and training (38). The Ontario *Emergency Preparedness Guide for People with Disabilities/Special Needs* did not mention people with intellectual disabilities, and their representative organisations were not involved in its development despite the Ontario government’s extensive consultation with other organisations of persons with disabilities, which meant it was not accessible to them (30). Despite this lack of inclusion, self-advocates with intellectual disabilities interviewed demonstrated a ‘mix of active participation and need for support in preparing for, and responding to, an emergency’ (30). Regular rehearsals of what to do in an emergency can help children and adults with intellectual disabilities to cope during them (30, 36, 37).

Social supports are incredibly important for people with intellectual disabilities’ ability to cope with different crises situations; however due to their social exclusion and lack of community integration, these supports are not always available to them (30). Being involved in preparedness activities is especially important for the increasing numbers of people with intellectual disabilities living independently, who do not have the support of the administrators of congregate care facilities to prepare for crises (27).

In Iran, school textbooks aimed at children with intellectual disabilities in different grades cover natural hazards, as well as preparedness, safety tips^4^, psychosocial support, and the emergency and relief organizations operating in Iran (with accompanying images for ease of understanding) (28). However, there was found to be a need for more content about sheltering in disasters, reunification, as well as disasters’ response and recovery to help children with intellectual disabilities to cope better during and after disasters (28). In Japan, an earthquake preparedness programme in special schools found that after the programme, most children with mild intellectual disabilities were able to take action to protect themselves without the teacher’s instruction, while those with more severe intellectual disabilities who were unable to take response actions on their own, no longer panicked when they heard the earthquake early warning sound and displayed greater receptivity to seek support from others (36). In Vietnam, however, inaccessible schools impacted on people with intellectual disabilities’ access to risk information (32).

#### 3.1.4 Exclusion from response

Like other people with disabilities, people with intellectual disabilities basic needs are not being met by humanitarian assistance or national government emergency response programmes, leading to a deterioration in their physical and mental well-being (15, 17, 18, 20, 26, 31, 39). Where such response programmes focus of people with disabilities, they often do not focus on the specific needs of people with intellectual disabilities (15). Challenging emergency and humanitarian environments can magnify the risks people with intellectual disabilities face and the nature of their impairment can make it hard for them to cope with the experiences they have during the response, resulting in challenging behaviours (15, 19, 20, 31, 39). Changes in their behaviour can lead to responses from caregivers which are inappropriate and increase their risk of exploitation, violence, and abuse (40).

People with intellectual disabilities may struggle to understand evacuation-related information during emergencies (22, 26). They may also become distressed during emergency situations and emergency-related stimuli, such as sirens/bells, fire drills, flashing lights, strangers, and emergency personnel, can ‘impede rather than support appropriate evacuation behaviors in individuals with intellectual disabilities’ (17, 18, 26, 29, 36). During rapid evacuations important disability-related items such as medications, sensory support, and assistive devices may be left behind (20, 21).

Shelters are challenging for people with intellectual disabilities and their families and they may experience stigma and discrimination from others in the shelters (17, 18, 20, 41). In Vietnam, the families caring for children or adults with intellectual disabilities who have problems controlling defecation or urination, or aggressive behavioural issues, were reluctant to take their children to safe places during disasters such as communal evacuation centres or neighbouring houses (32). In Vanuatu, safeguarding concerns, a desire for privacy, and disability stigma and discrimination also meant caregivers of women with intellectual disabilities choose not to go to evacuation centres or other communal evacuation gathering places during cyclones (42). In the US and Japan, group shelters were found to be overwhelming for children with intellectual disabilities, and parents were also worried about their behaviour and other people’s attitudes as a result (21, 31).

Special diets or selective eating can be threatening when food supplies are restricted, and limited food and water supplies are difficult for those with tendencies to use-up food and drink supplies as they do not understand the need for restrictions (20, 31). Limited access to specialists, rehabilitation centres, and general medical care and medicine during the response to crises can aggravate people with intellectual disabilities existing vulnerabilities and worsen their impairments (17, 43). The move to telehealth during Covid-19 was inaccessible for some people with intellectual disabilities, although others benefited from it (44).

Social distancing and isolation measures during Covid-19 were challenging for some children and adults with intellectual disabilities, due to difficulties related to understanding, communicating, and coping with such changes due to the strong need of routine/sameness and low adaptive skills, which had a negative impact on loneliness, their mental health and behaviour (10, 25, 40, 45). Children with intellectual disabilities were amongst those most affected by the disruption to schools (46). People with intellectual disabilities with technological skills may have had an advantage over people with intellectual disabilities who did not, as they could switch some of their activities from in-person to virtual, leaving them less socially isolated (40, 47).

However, there are some efforts to meet the specific needs of people with intellectual disabilities in humanitarian responses. For example, a menstrual health intervention for women and girls with intellectual disabilities and their caregivers using accessible materials in Vanuatu’s humanitarian setting responded to the risks they face from inadequate menstrual health in emergencies, which reduced evacuation options due to safety and privacy concerns (42, 48).

#### 3.1.5 Post crises

Post-crises contexts are often in disarray and unfamiliar, resulting in people with intellectual disabilities who were able to function independently prior to the crisis, losing their quality of life, independence and needing support to cope with these new contexts (15, 26, 43). Priorities post-crisis can focus those who have become disabled as a result of the crisis, leaving people with intellectual disabilities left out of rebuilding efforts (15, 16). Disruptions caused by crises, such as the conflict in Ukraine, can risk halting de-institutionalisation progress, forcing people with intellectual disabilities back into large residential facilities (17).

In the aftermath of crises, people with intellectual disabilities may experience significant declines in communication, behaviour, socialization, and other skills, which can make it more difficult for themselves and their caregivers (21–23, 25, 26, 31, 49, 50). The damage caused by disasters meant changes to routines which some children with intellectual disabilities find hard to cope with and some found it difficult to understand that their house or other familiar spaces had been destroyed (21, 22). Needing to relocate to unfamiliar areas can expose people with intellectual disabilities to increased stigma (15, 20, 21). They can face significant barriers to their long-term recovery (43, 51). Their access to medication and rehabilitation services can be disrupted, making life harder (15, 17, 26, 43, 49). Children with intellectual disabilities who can no longer attend school during or after the crisis, can lose the progress they had been making^5^ (20, 22). Adults and children with intellectual disabilities may also lose their family members or carers who provided them with essential support due to death, injury or displacement, thus requiring a new support structure and support for their grief (20, 26). People with intellectual disabilities’ support requirements sometimes increase after crises, meaning family caregivers are less able to work and recover from the crisis (42).

Experiences of crises can be extremely traumatic and distressing for people with intellectual disabilities, contributing to their grief, loss, and sadness (18, 19, 21–23, 25). Clinicians reported increased use of antipsychotics, antidepressants, mood stabilisers and benzodiazepines among their patients with intellectual disabilities as a result of the Covid-19 pandemic (23). Due to a higher risk of previous traumas prior to the crisis, people with intellectual disabilities are at higher risk of post-traumatic stress disorder (PTSD) after crises (18, 51, 53). PTSD can present itself differently in individuals with intellectual disabilities, and those working with them need to recognize these PTSD symptoms (such as aggression, anger outbursts, self-injuries, and non-compliance) (51). Trauma can sometimes also lead to increased incontinence of some people with intellectual disabilities (21, 22, 42).

A study post-earthquake in Italy found that the resiliency and the recovery of pre-disaster functioning in children with intellectual disabilities mainly depended on their immediate inclusion in routine, intensive rehabilitation programmes and the steadying, as far as possible, of daily routines (49).

## 4 Discussion

The importance of taking into account the experiences of people with disabilities in different crises contexts is increasingly recognised due to the disproportionate impact conflicts, natural hazard related disasters, and public health emergencies have on them (6, 7), and this review shows that there is an increasing evidence base around the specific experiences of people with intellectual disabilities in crises. 29 of the 40 studies have been published since 2020. Knowledge about the experiences of crises affected people with intellectual disabilities is not equally divided. Most of the focus of the available literature on the experiences of people with intellectual disabilities explored their experiences of disasters (n=21), and within that there was a large focus on earthquakes (n= 9); followed by public health emergencies (exclusively Covid-19 focused) (n=10)^6^; conflict (n=5)^7^; and some focusing on multiple crises (n=4). While there is a mix from high-, middle- and low-income countries, many of the studies focus on the same few high-income countries (Japan, Italy, Türkiye, United States (n=18)). 13 of the studies focused on the experiences of children with intellectual disabilities. Despite people with intellectual disabilities not being homogenous, few studies clarified the level of support needs of the individuals with intellectual disabilities involved, although two did have a focus on those with very high support needs. Going forward, more needs to be learnt about children and adults with intellectual disabilities’, with different levels of support needs (especially those with profound and multiple intellectual disabilities), experiences of different types of crises and in a wider variety of contexts.

There are aspects of intellectual impairment which make experiences of crises more challenging and place people with intellectual disabilities at greater risk such as communication and cognitive challenges (including judgement and reasoning skills); limited coping skills; need for structure and familiarity; impulsivity and difficulty recognising danger and dependency on support. Some people with intellectual disabilities experience other conditions, for example, autism or epilepsy, which could have further implications for their vulnerability in crises. However, their disproportionately negative experiences of crises are not solely due to any inherent vulnerability. In line with other literature about groups who are considered vulnerable in crises, vulnerability is a consequence of a complex interaction of social structures and combination of social, physical, environmental, cultural and political factors, rather than solely an essential constituent of intellectual disability (16, 55, 56). As a result, while people with intellectual disabilities are disproportionally vulnerable in crises and find it harder to cope, they are less vulnerable when there is a more inclusive society, suitable support structures around them, and their lives are valued and understood.

Disability stigma can result in the perception that people with intellectual disabilities life is worth less than others, and therefore they are more likely to die and be a lower priority in preparedness and response efforts as their life is not valued equally to others on the ground in crises (7, 15, 16, 39). Their social exclusion contributes to responders’ lack of familiarity with them, their communication, behaviour and needs, which has negative consequences in crises. It also shrinks their social networks, which in turn are disrupted during crises, further shrinking the support available to them (7). Disability related stigma can isolate caregivers too and make it harder for them to access the services for the children and adults with intellectual disabilities they support, increasing the burden of care on them (39). The papers highlight the impact crises have on the caregivers of people with intellectual disabilities, making it more difficult, stressful, and burdensome (17, 21–23, 31, 39, 50, 53, 57). This in turn has negative impacts on the people with intellectual disabilities who rely on them during and after crises (18, 50). Building up a wider support network of families, professional staff, and lay citizens who value and can help people with intellectual disabilities to respond to crises is important (18, 41).

People with intellectual disabilities’ barriers to inclusive education and lack of inclusion in preparedness activities are another factor contributing to their negative experience in crises. Their capacities to cope in crises increase the more they are included. Preparedness trainings aimed at people with intellectual disabilities and tailored to their communication needs have been found to be effective (27, 36–38, 58). For example, an earthquake preparedness course in Turky resulted in children with intellectual disabilities independently exhibiting the drop-cover-hold move and sharing their locations through the personal safety app during two real earthquakes after the conclusion of their training (37).

## 5 Conclusion

People with intellectual disabilities who are crisis affected, fare worse than people with intellectual disabilities who have been unaffected by crises (49). They are more traumatised, dependent, vulnerable, have worse health, education, and a poorer quality of life, and weaker support systems around them. Yet it does not have to be this way and if they are considered and included in crises preparedness, response and recovery efforts they will not be so disproportionally affected by conflicts, natural hazard related disasters, and public health emergencies.

## Footnotes

1 Due to time limitations for this additional review, only Web of Science was searched.

2 In general, people with disabilities were more than twice as likely to die from COVID-19 than their peers without disabilities (24).

3 For people with intellectual disabilities living in institutions, like many in Ukraine, the loss of personnel as a result of the war meant they often had little help or support (17).

4 Including one example in relation to the increased risk of abuse that exist in crises contexts and how to say no (28).

5 In addition, in the UK, children with intellectual disabilities who learnt from home during school closures during Covid-19, had increased rates of subsequent school absence (52).

6 Studies of people with disabilities in other epidemics such as Ebola did not include people with intellectual disabilities (54).

7 Despite calls for more evidence about the experiences of conflict-affected people with intellectual disabilities, the evidence base is still smaller than that of other crises, even with the large number of conflict-affected populations across the world (15, 16).
